# Effects of traditional Chinese mind-body exercises for patients with chronic fatigue syndrome: A systematic review and meta-analysis

**DOI:** 10.7189/jogh.13.04157

**Published:** 2023-11-24

**Authors:** Lingjun Kong, Jun Ren, Sitong Fang, Yunlong Li, Zhiwei Wu, Xin Zhou, Qiukui Hao, Min Fang, Yu-Qing Zhang

**Affiliations:** 1 Department of Tuina, Shuguang Hospital, Shanghai University of Traditional Chinese Medicine, Shanghai, China; 2 Yueyang Hospital of Integrated Traditional Chinese and Western Medicine, Shanghai University of Traditional Chinese Medicine, Shanghai, China; 3 Institute of Tuina, Shanghai Institute of Traditional Chinese Medicine, Shanghai, China; 4 School of Rehabilitation Science, McMaster University, Hamilton, Ontario, Canada; 5 CEBIM (Center for Evidence Based Integrative Medicine) – Clarity Collaboration, Guang'anmen Hospital, China Academy of Chinese Medical Sciences, Beijing, China; 6 Department of Health Research Methods, Evidence, and Impact, McMaster University, Hamilton, Ontario, Canada; 7 Institute of Acupuncture and Moxibustion, China Academy of Chinese Medical Sciences, Beijing, China

## Abstract

**Background:**

Chronic fatigue syndrome (CFS) is a global public health concern. We performed this systematic review of randomised controlled trials (RCTs) to evaluate the effects and safety of traditional Chinese mind-body exercises (TCME) for patients with CFS.

**Methods:**

We comprehensively searched MEDLINE, Embase, Web of Science, PsycINFO, Cochrane Library, CNKI, VIP databases, and Wanfang Data from inception to October 2022 for eligible RCTs of TCME for CFS management. We used Cochran’s Q statistic and *I*^2^ to assess heterogeneity and conducted subgroup analyses based on different types of TCME, background therapy, and types of fatigue. We also assessed the quality of evidence using the Grading of Recommendations, Assessment, Development, and Evaluations (GRADE) approach.

**Results:**

We included 13 studies (n = 1187) with a maximal follow-up of 12 weeks. TCME included Qigong and Tai Chi. At the end of the treatment, compared with passive control, TCME probably reduces the severity of fatigue (standardised mean differences (SMD) = 0.85; 95% confidence interval (CI) = 0.64, 1.07, moderate certainty), depression (SMD = 0.53; 95% CI = 0.34, 0.72, moderate certainty), anxiety (SMD = 0.29; 95% CI = 0.11, 0.48, moderate certainty), sleep quality (SMD = 0.34; 95% CI = 0.10, 0.57, low certainty) and mental functioning (SMD = 0.90; 95% CI = 0.50, 1.29, low certainty). Compared with other active control therapies, TCME results in little to no difference in the severity of fatigue (SMD = 0.08; 95% CI = -0.18, 0.34, low certainty). For long-term outcomes, TCME may improve anxiety (SMD = 1.74; 95% CI = 0.44, 3.03, low certainty) compared to passive control. We did not identify TCME-related serious adverse events.

**Conclusions:**

In patients with CFS, TCME probably reduces post-intervention fatigue, depression, and anxiety and may improve sleep quality and mental function compared with passive control, but has limited long-term effects. These findings will help health professionals and patients with better clinical decision-making.

**Registration:**

PROSPERO: CRD42022329157.

Chronic fatigue syndrome (CFS) is a debilitating condition characterised by excessive fatigue, unrefreshing sleep, malaise, anxiety, and depression, without a known cause or curative treatment [1,2]. A 2020 systematic review estimated a 0.89% CFS global prevalence according to a definition provided by the Centers for Disease Control and Prevention (CDC) in 1994 [3]. Over 80% of CFS patients experience long-term symptoms that lead to a substantial disability in carrying out normal work activities [4]. In the USA, CFS results in a 37% and 54% annual decrease in household and labor force productivity, respectively, bringing an annual loss of US$9.1 billion [5]. It also presents a significant economic burden for patients and their families [6,7].

As of yet, pharmacological interventions have suboptimal effects due to CFS’ unclear etiology and pathophysiology. Given their long-term adverse effects and financial burden [8,9], many patients seek complementary and alternative therapy to improve their symptoms and quality of life [10]. For CFS, a standard treatment program usually involves exercise therapy, which, according to a Cochrane systematic review, might improve CFS patients’ fatigue, sleep disturbance, physical function, and self-perceived general health [11]. However, this review excluded traditional Chinese mind-body exercises (TCME) such as Tai Chi and Qigong.

TCME emphasises the coordination and unification of breathing, mental relaxation, and body movements to improve physical and mental health status [12], with clinicians recommending Tai Chi and Qigong for managing CFS symptoms [13-15]. Researchers have shown its potential improvements in fatigue, anxiety, depression, and quality of sleep [16-19]. However, previous reviews of mind-body interventions for CFS included few studies addressing the effectiveness of TCME for patients with CFS, as they did not include Chinese literature databases [20]. A systematic review and meta-analysis of TCME for CFS did not show trustworthy evidence due to its inappropriate searching strategy and pooling analysis [21]. To address these gaps, we conducted a systematic review to assess the effects and safety of TCME for CFS management.

## METHODS

We reported this systematic review following the Preferred Reporting Items for Systematic Reviews and Meta-analyses (PRISMA) statement [22] and registered it in the International Prospective Register of Systematic Reviews (PROSPERO) (CRD42022329157).

### Search strategy

We designed a search strategy with the help of a medical librarian for MEDLINE, Embase, Web of Science, PsycINFO, Cochrane Library, China National Knowledge Infrastructure (CNKI), Wanfang Data Information, and Chinese Science and Technical Periodical Databases (VIP), searching them from inception to October 2022, without restrictions to language or publication status (**Online Supplementary Documen**t). We also screened the reference lists of the related reviews and clinical guidelines to identify eligible studies.

### Study selection

Two reviewers independently reviewed the literature according to pre-defined inclusion and exclusion criteria. We included randomised controlled trials (RCTs) examining the efficiency of TCME for CFS among patients diagnosed by any valid diagnostic criteria, without limitations on age, gender, or nationality. Our intervention of interest was TCME including Tai Chi, Qigong alone or other TCME types (defined as a combination of physical and mental training) with or without the background therapy. Our comparators were passive (waiting list, no treatment, and minimal education) or active controls (pharmacological therapy, cognitive behavior therapy, acupuncture, moxibustion, point application, and other exercises therapy without mind-body exercises), compared as TCME vs passive/active control, TCME plus active control vs active control only, or similarly in a RCT. Our outcomes of interest were any of the following: fatigue, sleep quality, anxiety, depression, and quality of life measured using any validated scale and adverse events.

### Data extraction

Using a pre-designed form, reviewers independently extracted data on study characteristics (first author, author information, publication year, and country where researchers conducted the study), participant characteristics (sample size and mean age), interventions, and outcomes (type of interventions and comparators, length of intervention, main outcomes, and follow-up duration). We resolved discrepancies by discussion among all reviewers.

### Risk of bias assessment

Two reviewers independently evaluated the quality of the eligible studies using Cochrane's Risk of Bias tool for RCTs [23] per the following items: random sequence generation, allocation concealment, blinding of participants and personnel, incomplete outcome data, selective reporting, and other biases, with each categorised as having “low”, “unclear”, and “high” risk of bias. We resolved discrepancies by discussion, when necessary, with adjudication by a third reviewer.

### Statistical analysis

We used RevMan, version 5.3 (The Cochrane Collaboration, Oxford, United Kingdom) to perform the meta-analysis. We calculated standardised mean differences (SMD) and 95% confidence intervals (CIs) for continuous variables measured by different measure instruments based on the mean changes between pre- and post-treatments and used a random effects models to pool the data. To assess heterogeneity, we used the Cochran’s Q statistic (considered to be statistically significant when *P* < 0.10) and *I*^2^ (where *I*^2^>30% indicated moderate heterogeneity, *I*^2^>50% substantial heterogeneity, and *I*^2^>75% considerable heterogeneity). We then conducted subgroup analyses based on different types of TCME, background therapy, and types of fatigue. If there were more than ten studies, we used the funnel plot to assess the possibilities of publication bias. For the meta-analysis, we considered *P* < 0.05 as statistically significant. In addition, we assessed the overall quality of evidence using the Grading of Recommendations, Assessment, Development, and Evaluations (GRADE) framework by considering the risk of bias, inconsistency, indirectness, imprecision, and publication bias.

### Equity, diversity and inclusion statement

The author team was gender balanced and consists of junior, mid-career, and senior researchers from different disciplines and two countries. Our study population included both male and female subjects from different socioeconomic and cultural backgrounds.

### Patient and public involvement

Patients and/or the public were not involved in the design, or conduct, or reporting, or dissemination plans of this research.

### Ethical approval

We did not require ethical approval, as we used secondary data from previously published studies which obtained informed consent from participants.

## RESULTS

**Figure 1:**
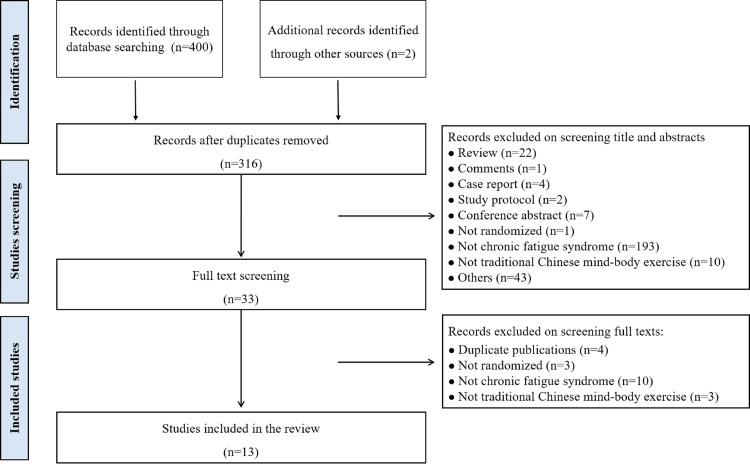
Flowchart of study selection.

We identified 402 records through the literature search, with 316 remaining after deduplication. We excluded 283 studies after screening the titles and abstracts and a further 20 after full-text screening, leaving 13 studies for inclusion in our review [16-19,24-32] (**Figure 1** and Table S1 in the **Online Supplementary Document**).

### Study characteristics

We included 13 studies with 1187 participants, with a mean age of 40.33 (standard deviation (SD) = 4.36 years) [16-19,24-32], all from China, conducted between 2012 and 2022. TCME included Qigong (n = 10) [16-19,24,26-29,31,32] and Tai Chi (n = 3) [25,30,32]. Six studies compared TCME with passive control [16-19,28,31], another six compared TCME plus background therapy with the same background therapy [25-27,29,30,32], and three compared TCME with active control interventions including cognitive behavioral therapy [24], moxibustion [27], and point application [29]. The length of treatment varied between four and 16 weeks, while exercise duration ranged between 20 and 120 minutes (mean = 43.75 (SD = 24.16)). The common intensity of Qigong exercises was at least 30-minute/session, more than three sessions/week, and at least nine weeks. The longest follow-up time was 12 weeks. Patient important outcomes included fatigue, anxiety, depression, quality of life, and sleep quality (**Table 1**).

**Table 1:** Study characteristics

First author, country (year)	Diseases	Sample size, TCME/control	Mean age in years (standard deviation)	Duration	Follow-up	Main outcome assessments	Experimental group intervention	Control group intervention
Xie, China (2022) [24]	CFS	45/ 44	37.94 (11.34), 37.34 (9.86)	12 weeks	NR	MFI-20, PSQI, HADS	Qigong (30 min/session, 84 sessions)	Cognitive behavioral therapy (30 min/session, 84 sessions)
Chen, China (2021) [32]	CFS	34/36	50.68 (8.99), 51.21 (9.13)	4 weeks	NR	SF-36, MFI-20, 5-HT, NPY, SP, CGRP	Tai Chi (30 min/session, 56 sessions) plus conventional treatment	Conventional treatment
Zhang, China (2020) [25]	CFS	39/39	37.62 (10.02), 37.70(9.45)	8 weeks	NR	ChFS	Tai Chi (30-40 min/session, 32 sessions) plus herbal medicine (bid, 56 d)	Herbal medicine (tid, 56 d)
Lu, China (2019) [27]	CFS	24/23	37.18 (9.05), 38.92(7.69)	12 weeks	NR	ChFS	Qigong (30 min/session, 60 sessions) plus acupuncture (42 sessions)	Acupuncture (42 sessions)
Chan, 2017, China [17]	CFS	46/62	39.50, 42.00	9 weeks	12 weeks	HADS, Plasma adiponectin	Qigong (90 min/session, 16 sessions)	Waiting list
Na, China (2017) [27]	CFS	30/30	NR	4 weeks	NR	ChFS	Qigong (24 sessions)	Moxibustion (30-40 min/session, 24 sessions); thunder-fire moxibustion (30-40 min/session, 24 sessions); thunder-fire moxibustion (30-40 min/session, 24 sessions) plus Qigong (24 sessions)
Yin, China (2016) [28]	CFS	27/30	33.13 (9.11), 32.70 (9.47)	12 weeks	NR	MFI-20, PSQI, SF-36	Qigong (30-120 min/session, 72 sessions)	Minimal education (120 min/session, 12 sessions)
Li, China (2015) [16]	CFS	22/24	43.60 (7.59), 44.06 (4.88)	12 weeks	NR	ChFS, SF-12, HADS, BMSWBI-S	Qigong (120 min/session, 10 sessions, five weeks 15-30 min/session, ≥3 sessions/week)	Waiting list
Chan, China (2014) [18]	CFS	75/75	39.10 (7.80), 38.90 (8.01)	9 weeks	12 weeks	ChFS, HADS, PSQI	Qigong (90 min/session, 16 sessions)	Waiting list
Yu, China (2014) [29]	CFS	42/42	39.48 (7.56), 40.70 (6.73), 41.31 (6.12), 42.65 (6.68)	40 d	NR	ChFS, PSQI	Qigong (30 min/session, 40 sessions)	Qigong (30 min/session, 40 sessions) plus point application (6-12 h/session, 20 sessions); point application (6-12 h/session, 20 sessions); medicine (40 mg, tid, 40 d)
Chan, China (2013) [19]	CFS	72/65	42.40 (6.70), 42.50 (6.40)	16 weeks	NR	ChFS, HADS	Qigong (120 min/session, 10 sessions, five weeks ≥30 min/session, ≥3 sessions/week)	Waiting list
Lin, China (2013) [30]	CFS	27/26	35.90 (10.20), 34.90 (12.80)	4 weeks	NR	FAI, SF-36	Tai Chi (20-30 min/session, 28 sessions) plus acupuncture (30 min/session, 12 sessions)	Acupuncture (30 min/session, 12 sessions)
Ho, China (2012) [31]	CFS	33/31	42.10 (7.30), 42.50 (5.50)	16 weeks	NR	ChFS, SF-12, Telomerase Activity	Qigong (120 min/session, 10 sessions, five weeks, 30 min/session, 84 sessions)	Waiting list

TCME – traditional Chinese mind-body exercises, CFS – chronic fatigue syndrome, ChFS – Chalder fatigue scale, HADS – hospital anxiety and depression scale, MFI-20 – multidimensional fatigue inventory-20, SF-36 – short form 36-questionnaire, SF-12 – medical outcomes study 12-item short-form health survey, PSQI – Pittsburgh sleep quality index, BMSWBI-S – body-mind-spirit well-being inventory, 5-HT – 5-hydroxytryptamine, FAI – fatigue assessment instrument, NPY – neuropeptide Y, SP – substance P, CGRP – alcitonin gene-related peptide, NR – no report, bid – bis in die, tid – ter in die

### Risk of bias

Eleven trials [16-19,24-28,31,32] appropriately generated the random sequence using random number tables or computers. Only four [24,27,28,31] used adequate allocation concealment methods, which was unclear in the remaining seven [16-19,25,26,29-32]. None of the studies reported blinding of participants. One study [26] used an independent outcome assessor, and the others were unclear. Nine studies [16-19,25,27,29,31,32] analysed data using all intention-to-treat (ITT) or modified ITT analysis. Only one study[24] was categorised as having a high risk of bias in selective reporting outcome, and the others were at low risk of bias (Figure S1 in the **Online Supplementary Document**). We assessed the overall quality of evidence using the GRADE method (**Table 2**).

**Table 2:** The quality of evidence by GRADE

	Certainty assessment						Summary of finding			
**Disease/condition**	**n of studies**	**Study design**	**Risk of bias**	**Inconsistency**	**Indirectness**	**Imprecision**	**Other considerations**	**n of patients, TCME/control therapy**	**Effect as SMD (absolute 95% CI)**	**Certainty**
Traditional Chinese mind-body exercises compared with passive control for CFS										
*Fatigue*	11	RCT	Serious*	Serious†	Not serious	Not serious	None	425/421	0.85 (0.64, 1.07)	Moderate
*Anxiety*	4	RCT	Serious*	Not serious	Not serious	Not serious	None	215/226	0.29 (0.11, 0.48)	Moderate
*Depression*	4	RCT	Serious*	Not serious	Not serious	Not serious	None	215/226	0.53 (0.34, 0.72)	Moderate
*Sleep quality*	3	RCT	Serious*	Not serious	Not serious	Serious^‡^	None	144/147	0.32 (0.05, 0.60)	Low
*Physical functioning*	2	RCT	Serious*	Not serious	Not serious	Serious‡	None	55/55	0.11 (-0.26, 0.49)	Low
*Mental functioning*	2	RCT	Serious*	Not serious	Not serious	Serious‡	None	55/55	0.90 (0.50, 1.29)	Low
Traditional Chinese mind-body exercises alone compared with active control for CFS										
*Fatigue*	3	RCT	Serious*	Not serious	Not serious	Serious‡	none	117/116	0.08 (-0.18, 0.34)	Low

TCME – traditional Chinese mind-body exercises, CFS – chronic fatigue syndrome, CI – confidence interval, SMD – standardised mean difference, RCT – randomized controlled trial

*There were serious limitations in allocation concealment and blinding.

†The test for heterogeneity is significant (*I^2^*>50%).

‡Small sample size less than 400.

### Effectiveness of TCME on post-intervention outcomes

#### TCME vs passive control

##### Fatigue

**Figure 2:**
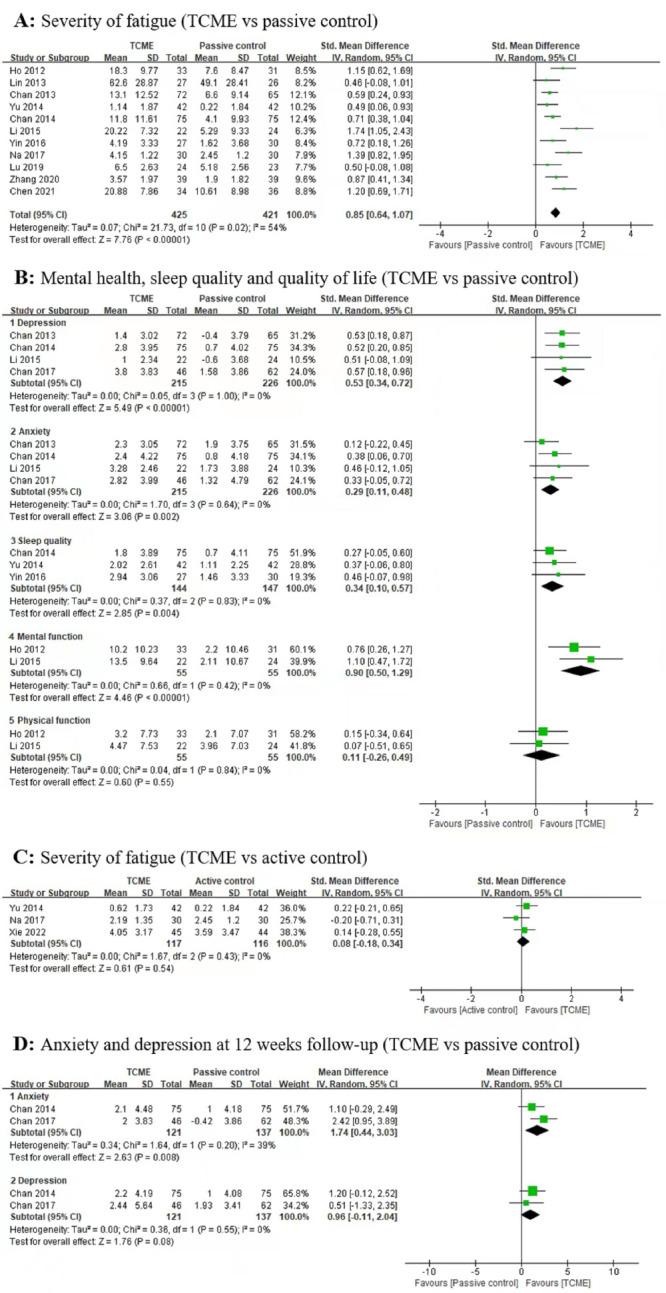
Effects of TCME for patients with chronic fatigue syndrome. TCME – traditional Chinese mind-body exercises.

We identified 11 RCTs [16,18,19,25-32] with 960 CFS patients comparing TCME with passive control on severity of fatigue, using Qigong and Tai Chi as TCME interventions. The meta-analysis indicated that TCME probably results in a large reduction in the severity of post-intervention fatigue (SMD = 0.85; 95% CI = 0.64, 1.07, *I*^2^ = 54%, moderate certainty) (**Figure 2**).

##### Mental health

Four studies [16-19] with 441 participants measured the effects of TCME on anxiety and depression compared with passive control, all using Qigong as TCME intervention. Compared with the passive control, TCME probably reduced post-intervention depression (SMD = 0.53; 95% CI = 0.34, 0.72; *I*^2^ = 0%, moderate certainty), and anxiety (SMD = 0.29; 95% CI = 0.11, 0.48; *I*^2^ = 0%, moderate certainty) (**Figure 2**).

##### Sleep quality

Three studies [18,28,29] with 291 participants assessed the effects of TCME using Qigong on sleep quality comparing with passive control. We found TCME may improve post-intervention sleep quality (SMD = 0.34; 95% CI = 0.10, 0.57; *I*^2^ = 0%, low certainty) (**Figure 2**).

##### Quality of life

Two studies [16,31] with 110 participants assessed the effects of TCME on quality of life compared with passive control, using Qigong as the TCME intervention. TCME may result in a large improvement in post-intervention mental function (SMD = 0.90; 95% CI = 0.50, 1.29; *I*^2^ = 0%, low certainty), but did not improve post-intervention physical function (SMD = 0.11; 95% CI = -0.26, 0.49; *I*^2^ = 0%, low certainty) (**Figure 2**).

#### TCME vs active control

Three studies [24,27,29] with 233 patients compared the effects of TCME via Qigong with active control. The active control interventions included cognitive behavioral therapy, moxibustion, and point application. The meta-analysis of three trials indicated that compared to other active control, TCME may result in little to no difference in the severity of post-interventions fatigue (SMD = 0.08; 95% CI = -0.18, 0.34; *I*^2^ = 0%, low certainty) (**Figure 2**) [25,26,28].

#### Effectiveness of TCME on outcomes at 12-week Follow-up

Only one study [18] with 115 participants reported that, compared to passive control, TCME may improve fatigue with a statistically significant difference (mean change on Chalder Fatigue Scale = 12.2 vs 5.3, *P* < 0.001), but the effectiveness of TCME on sleep quality was not statistically significant (mean change on Pittsburgh Sleep Quality Index = 1.7 vs 0.9, *P* = 0.064). Meanwhile, two studies [17,18] with 258 participants reported the effectiveness of TCME compared to the passive control at 12 weeks follow-up. Our pooled analysis showed that TCME may result in a large reduction in anxiety (SMD = 1.74; 95% CI = 0.44, 3.03; *I*^2^ = 39%, low certainty), but in little to no difference in depression (SMD = 0.96; 95% CI = -0.11, 2.04; *I*^2^ = 0%, low certainty) (**Figure 2**) compared with passive control.

### Adverse events

One study [18] with 150 participants reported that the most common adverse event is muscle ache, followed by palpitation, giddiness, knee pain, back ache, and dizziness. Xie et al. [24] reported mild muscle ache probably related to Qigong exercises, but without statistically significant differences in adverse events between Qigong exercise and cognitive behavioral therapy (*P* = 0.096).

### Subgroup analysis

We conducted subgroup analyses to explore the possible impact of different background therapy, types of TCME, and types of fatigue. We found no subgroup effects on different types of the background therapy (TCME with background therapy vs without background therapy: *P* for interaction = 0.69, pharmacological vs non-pharmacological intervention: *P* for interaction = 0.88), types of TCME (Qigong vs Tai Chi: *P* for interaction = 0.83), or types of fatigue (physical vs mental: *P* for interaction = 0.16) (Figure S2-S5 in the **Online Supplementary Document**).

### Publication bias

**Figure 3:**
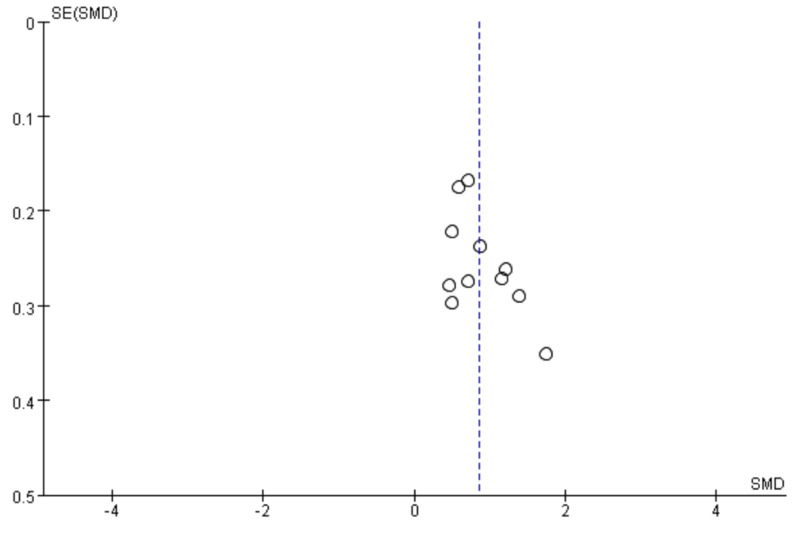
Funnel plot of the effect size of TCME on severity of fatigue in patients with chronic fatigue syndrome compared with passive control. TCME – traditional Chinese mind-body exercises.

The funnel plot of the effect size of TCME on fatigue was approximately symmetric, indicating that there was no publication bias (**Figure 3**).

## DISCUSSION

We evaluated the effectiveness and safety of TCME (Qigong and Tai Chi) for CFS. For post-intervention outcomes compared with passive control, moderate certainty evidence suggests that TCME probably reduce the severity of fatigue, depression, and anxiety, while low certainty evidence shows it may improve sleep quality and mental functioning. Low certainty evidence shows that TCME may result in little to no difference in the severity of post-interventions fatigue comparing to other active control therapies, yet its long-term effect is uncertain, as there is only low certainty evidence that TCME improves fatigue and anxiety at 12 weeks follow-up for CFS patients. TCME are generally safe due to the rare reports of minor adverse events.

A previous study reported that CFS’ common comorbidities include negative emotions such as depression or anxiety [33] and that it may be effective in improving physical and psychological syndromes by incorporating breathing, body movements, and mental relaxation to enhance self-awareness and well-being [34]. TCME has been shown to increase oxygen intake and improve the body’s hypoxia condition, thus reducing fatigue [13,35], while also having the potential to alter the sympathetic-adrenal-medullary and the hypothalamus-pituitary-adrenal axis by increasing activity in the parasympathetic nervous system, thus decreasing negative emotions [36]. Researchers have hypothesised that TCME may induce the release of neurotransmitters (dopamine, serotonin, and adrenaline) in the brain, which is associated with improvements in anxiety and depression [37,38]. Another study reported that TCME could increase pleasant mood by inhibiting the activity of the left hemisphere and promoting the excitation of the right hemisphere [39]. We also found moderate certainty evidence supporting that TCME probably reduces depression (post-intervention) and anxiety (post-intervention and at 12 weeks follow-up) in CFS patients.

Our findings are in line with existing systematic reviews. For example, one review that mind-body interventions may improve fatigue severity, as well as physical and mental functioning [21], but did not conduct a meta-analysis due to heterogeneity of interventions and outcomes. One meta-analysis included eight TCME studies’ effectiveness for CFS, and found improvement on fatigue and the sleep quality [21]. In their review, however, the authors neither conducted the pooled analysis on the severity of fatigue across studies reported with different instruments, nor subgroup analysis to explore sources of heterogeneity. Existing systematic reviews methodology quality is suboptimal.

### Strengths and limitations

Our review has several strengths, the first being a comprehensive search strategy which resulted in a 50% increase in the number of included studies compared with a previous systematic review [21]. Second, we conducted a meticulous synthesis to assess the effects of TCME compared with passive control or active control interventions, and performed subgroup analyses to explore the impact of types background therapy, TCME, and fatigue on the pooled effect estimates. Third, we assessed both the effectiveness of TCME on fatigue and on other patient-important outcomes including anxiety, depression, mental health, sleep quality, and quality of life.

Our review also has limitations. We conducted a post-hoc subgroup analysis, but could not explain the heterogeneity in fatigue outcome. Furthermore, all included studies were conducted in China, limiting generalisability, as geographical and contextual factors may result in variations in an unequal distribution of resources, ultimately affecting both patient care and outcomes. Future research could focus on recruiting CFS patients from various countries to address this issue, while simultaneously advancing fairness and accessibility in the global management of CFS.

### Implications

Future TCME RCTs should measure CFS’ long-term outcomes and objective outcomes (such as immune function status in CFS patients) conduct cost-benefit analysis, and evaluate its effects in CFS patients compared to other exercises (such as graded exercise therapy). Researchers should apply adequate allocation concealment methods and ensure outcome assessors are blinded. Future systematic review can explore the types, intensity, and duration of TCME on CFS’ treatment effect to find the most effective TCME in managing of CFS, while network meta-analyses could evaluate the effects of all CFS treatment, including TCME and other common strategies, such as pharmacological interventions, graded exercise therapy, or cognitive behavior therapy.

## CONCLUSIONS

TCME probably reduce post-intervention fatigue, depression, and anxiety, improves sleep quality and mental function, and causes minor adverse effect in patients with CFS when compared with passive control. However, data regarding its long-term effects on CFS are limited.

## Additional material


Online Supplementary Document
