# Supplementary material for: Effects of traditional Chinese mind-body exercises for patients with chronic fatigue syndrome: A systematic review and meta-analysis
Source: J Glob Health. 2023 Nov 24;13:04157. doi: 10.7189/jogh.13.04157 (PMC10666566; doi:10.7189/jogh.13.04157)
Supplement: Online Supplementary Document [file jogh-13-04157-s001.pdf]

Supplementary appendix

Supplement to:

Effects of traditional Chinese mind-body exercises for patients with chronic fatigue syndrome: a systematic review and meta-analysis

Full search strategy 1
Risk of bias assessment (Figure S1) 13
The results of subgroup analyses (Figure S2-S5) 14
Excluded studies with reasons for exclusion at full-text screening stage (Table S1) 16

Full search strategy

Search and strategy of traditional Chinese mind-body exercises for chronic fatigue syndrome

Medline (OVID)

Database: OVID Medline 1946 to 2022/09/30
Date:2022/10/02
Search Strategy:
----
1 Fatigue Syndrome, Chronic/
2 (chronic adj3 fatigue).mp. [mp=title, book title, abstract, original title, name of substance word, subject heading word, floating sub-heading word, keyword heading word, organism supplementary concept word, protocol supplementary concept word, rare disease supplementary concept word, unique identifier, synonyms]
3 (fatigue adj3 syndrome).mp. [mp=title, book title, abstract, original title, name of substance word, subject heading word, floating sub-heading word, keyword heading word, organism supplementary concept word, protocol supplementary concept word, rare disease supplementary concept word, unique identifier, synonyms]
4 (myalgic adj3 encephalomyelitis).mp. [mp=title, book title, abstract, original title, name of substance word, subject heading word, floating sub-heading word, keyword heading word, organism supplementary concept word, protocol supplementary concept word, rare disease supplementary concept word, unique identifier, synonyms]
5 CFS.mp.
6 Royal free disease.mp.
7 fatigue-fibromyalgia.mp.
8 (chronic adj3 mononucleosis).mp. [mp=title, book title, abstract, original title, name of substance word, subject heading word, floating sub-heading word, keyword heading word, organism supplementary concept word, protocol supplementary concept word, rare disease supplementary concept word, unique identifier, synonyms]
9 (chronic adj3 Epstein Barr).mp. [mp=title, book title, abstract, original title, name of substance word, subject heading word, floating sub-heading word, keyword heading word, organism supplementary concept word, protocol supplementary concept word, rare disease supplementary concept word, unique identifier, synonyms]
10 systemic exertion intolerance.mp.
11 epidemic neuromyasthenia.mp.
12 CFIDS.mp.

13	PVFS.mp.
14	Yuppie.mp.
15	iceland disease.mp.
16	akureyri disease.mp.
17	or/1-16
18	traditional Chinese exercises.mp.
19	mind-body exercises.mp.
20	(Tai Chi or tai ji or taijiquan or taichi).mp.
21	Qigong.mp.
22	(Five-animal exercises or Wuqinxi).mp.
23	Yijinjing.mp.
24	Baduanjin.mp.
25	or/18-24
26	randomized controlled trial.pt.
27	controlled clinical trial.pt.
28	randomi?ed.ab.
29	placebo.ab.
30	drug therapy.fs.
31	randomly.ab.
32	trial.ab.
33	groups.ab.
34	or/26-33
35	exp animals/ not humans.sh.
36	34 not 35
37	17 and 25 and 36

EMBASE (OVID)

Database: Embase <1974 to 2022/09/30>
Date: 2022/10/02
Search Strategy:
----
1 exp chronic fatigue syndrome/
2 (chronic adj3 fatigue).mp. [mp=title, abstract, heading word, drug trade name, original title, device manufacturer, drug manufacturer, device trade name, keyword heading word, floating subheading word, candidate term word]
3 (fatigue adj3 syndrome).mp. [mp=title, abstract, heading word, drug trade name, original title, device manufacturer, drug manufacturer, device trade name, keyword heading word, floating subheading word, candidate term word]
4 (myalgic adj3 encephalomyelitis).mp. [mp=title, abstract, heading word, drug trade name, original title, device manufacturer, drug manufacturer, device trade name, keyword heading word, floating subheading word, candidate term word]
5 CFS.mp.
6 Royal free disease.mp.
7 fatigue-fibromyalgia.mp.
8 (chronic adj3 mononucleosis).mp. [mp=title, abstract, heading word, drug trade name, original title, device manufacturer, drug manufacturer, device trade name, keyword heading word, floating subheading word, candidate term word]
9 (chronic adj3 Epstein Barr).mp. [mp=title, abstract, heading word, drug trade name, original title, device manufacturer, drug manufacturer, device trade name, keyword heading word, floating subheading word, candidate term word]
10 systemic exertion intolerance.mp.
11 epidemic neuromyasthenia.mp.
12 CFIDS.mp.
13 PVFS.mp.
14 Yuppie.mp.
15 iceland disease.mp.
16 akureyri disease.mp.
17 or/1-16
18 traditional Chinese exercises.mp.
19 mind-body exercises.mp.
20 (Tai Chi or tai ji or taijiquan or taichi).mp.
21 Qigong.mp.
22 (Five-animal exercises or Wuqinxi).mp.
23 Yijinjing.mp.
24 Baduanjin.mp.
25 or/18-24

26 randomized controlled trial/
27 Controlled clinical study/
28 random$.ti,ab.
29 randomization/
30 intermethod comparison/
31 placebo.ti,ab.
32 (compare or compared or comparison).ti.
33 ((evaluated or evaluate or evaluating or assessed or assess) and (compare or compared or comparing or comparison)).ab.
34 (open adj label).ti,ab.
35 ((double or single or doubly or singly) adj (blind or blinded or blindly)).ti,ab.
36 double blind procedure/
37 parallel group$1.ti,ab.
38 (crossover or cross over).ti,ab.
39 ((assign$ or match or matched or allocation) adj5 (alternate or group$1 or intervention$1 or patient$1 or subject$1 or participant$1)).ti,ab.
40 (assigned or allocated).ti,ab.
41 (controlled adj7 (study or design or trial)).ti,ab.
42 (volunteer or volunteers).ti,ab.
43 human experiment/
44 trial.ti.
45 or/19-37
46 (random$ adj sampl$ adj7 ("cross section$" or questionnaire$1 or survey$ or database$1)).ti,ab. not (comparative study/ or controlled study/ or randomi?ed controlled.ti,ab. or randomly assigned.ti,ab.)
47 Cross-sectional study/ not (randomized controlled trial/ or controlled clinical study/ or controlled study/ or randomi?ed controlled.ti,ab. or control group$1.ti,ab.)
48 (((case adj control$) and random$) not randomi?ed controlled).ti,ab.
49 (Systematic review not (trial or study)).ti.
50 (nonrandom$ not random$).ti,ab.
51 "Random field$".ti,ab.
52 (random cluster adj3 sampl$).ti,ab.
53 (review.ab. and review.pt.) not trial.ti.
54 "we searched".ab. and (review.ti. or review.pt.)
55 "update review".ab.
56 (databases adj4 searched).ab.
57 (rat or rats or mouse or mice or swine or porcine or murine or sheep or lambs or pigs or piglets or rabbit or rabbits or cat or cats or dog or dogs or cattle or bovine or monkey or monkeys or trout or marmoset$1).ti. and animal experiment/
58 Animal experiment/ not (human experiment/ or human/)
59 or/46-58
60 45 not 59

61	17 and 25 and 60

PsycInfo (OVID)

Database: APA PsycInfo <1806 to August Week 5 2022>
Date:2022/10/02
Search Strategy:
----
1 chronic fatigue syndrome/
2 (chronic adj3 fatigue).mp. [mp=title, abstract, heading word, table of contents, key concepts, original title, tests & measures, mesh word]
3 (fatigue adj3 syndrome).mp. [mp=title, abstract, heading word, table of contents, key concepts, original title, tests & measures, mesh word]
4 (myalgic adj3 encephalomyelitis).mp. [mp=title, abstract, heading word, table of contents, key concepts, original title, tests & measures, mesh word]
5 CFS.mp.
6 Royal free disease.mp.
7 fatigue-fibromyalgia.mp.
8 (chronic adj3 mononucleosis).mp. [mp=title, abstract, heading word, table of contents, key concepts, original title, tests & measures, mesh word]
9 (chronic adj3 Epstein Barr).mp. [mp=title, abstract, heading word, table of contents, key concepts, original title, tests & measures, mesh word]
10 systemic exertion intolerance.mp.
11 epidemic neuromyasthenia.mp.
12 CFIDS.mp.
13 PVFS.mp.
14 Yuppie.mp.
15 iceland disease.mp.
16 akureyri disease.mp.
17 or/1-16
18 traditional Chinese exercises.mp.
19 mind-body exercises.mp.
20 (Tai Chi or tai ji or taijiquan or taichi).mp.
21 Qigong.mp.
22 (Five-animal exercises or Wuqinxi).mp.
23 Yijinjing.mp.
24 Baduanjin.mp.
25 or/18-24
26 (double-blind or random: assigned or control).tw.
27 clinical trials/
28 (controlled adj3 trial*).mp. [mp=title, abstract, heading word, table of contents, key concepts, original title, tests & measures, mesh word]
29 (clinical adj2 trial*).mp. [mp=title, abstract, heading word, table of contents, key concepts, original title, tests & measures, mesh word]

30 (randomi?ed adj7 trial*).mp. [mp=title, abstract, heading word, table of contents, key concepts, original title, tests & measures, mesh word]

31 or/26-30

32 17 and 25 and 31

Cochrane Library

Search Name:
Date Run: 2022/10/02
Search Strategy:

#1 MeSH descriptor: [Fatigue Syndrome, Chronic] explode all trees
#2 chronic near/3 fatigue
#3 fatigue near/3 syndrome
#4 myalgic near/3 encephalomyelitis
#5 CFS
#6 Royal free disease
#7 fatigue-fibromyalgia
#8 chronic near/3 mononucleosis
#9 chronic near/3 Epstein Barr
#10 systemic exertion intolerance
#11 epidemic neuromyasthenia
#12 CFIDS
#13 PVFS
#14 Yuppie
#15 iceland disease
#16 akureyri disease
#17 #1 or #2 or #3 or #4 or #5 or #6 or #7 or #8 or #9 or #10 or #11 or #12 or #13 or #14 or #15 or #16
#18 traditional Chinese exercise
#19 mind-body exercise
#20 Tai Chi or tai ji or taijiquan or taichi
#21 Qigong
#22 Five-animal exercises or Wuqinxi
#23 Yijinjing
#24 Baduanjin
#25 #18 or #19 or #20 or #21 or #22 or #23 or #24
#25 #17 and #25 in Trials

Web of Science

Database:
Date:2022/10/02
Search Strategy:
----
27 #17 AND #25 AND #26
26 TS=(randomised OR randomized OR randomisation OR randomization OR placebo* OR (random* AND (allocat* OR assign*)) OR (blind* AND (single OR double OR treble OR triple))) NOT TS=(animal or animals or pisces or fish or fishes or catfish or catfishes or sheatfish or silurus or arius or heteropneustes or clarias or gariepinus or fathead minnow or fathead minnows or pimephales or promelas or cichlidae or trout or trouts or char or chars or salvelinus or salmo or oncorhynchus or guppy or guppies or millionfish or poecilia or goldfish or goldfishes or carassius or auratus or mullet or mullets or mugil or curema or shark or sharks or cod or cods or gadus or morhua or carp or carps or cyprinus or carpio or killifish or eel or eels or anguilla or zander or sander or lucioperca or stizostedion or turbot or turbots or psetta or flatfish or flatfishes or plaice or pleuronectes or platessa or tilapia or tilapias or oreochromis or sarotherodon or common sole or dover sole or solea or zebrafish or zebrafishes or danio or rerio or seabass or dicentrarchus or labrax or morone or lamprey or lampreys or petromyzon or pumpkinseed or pumpkinseeds or lepomis or gibbosus or herring or clupea or harengus or amphibia or amphibian or amphibians or anura or salientia or frog or frogs or rana or toad or toads or bufo or xenopus or laevis or bombina or epidalea or calamita or salamander or salamanders or newt or newts or triturus or reptilia or reptile or reptiles or bearded dragon or pogona or vitticeps or iguana or iguanas or lizard or lizards or anguis fragilis or turtle or turtles or snakes or snake or aves or bird or birds or quail or quails or coturnix or bobwhite or colinus or virginianus or poultry or poultries or fowl or fowls or chicken or chickens or gallus or zebra finch or taeniopygia or guttata or canary or canaries or serinus or canaria or parakeet or parakeets or grasskeet or parrot or parrots or psittacine or psittacines or shelduck or tadorna or goose or geese or branta or leucopsis or woodlark or lullula or flycatcher or ficedula or hypoleuca or dove or doves or geopelia or cuneata or duck or ducks or greylag or graylag or anser or harrier or circus pygargus or red knot or great knot or calidris or canutus or godwit or limosa or lapponica or meleagris or gallopavo or jackdaw or corvus or monedula or ruff or philomachus or pugnax or lapwing or peewit or plover or vanellus or swan or cygnus or columbianus or bewickii or gull or chroicocephalus or ridibundus or albifrons or great tit or parus or aythya or fuligula or streptopelia or risoria or spoonbill or platalea or leucorodia or blackbird or turdus or merula or blue tit or cyanistes or pigeon or pigeons or columba or pintail or anas or starling or sturnus or owl or athene noctua or pochard or ferina or cockatiel or nymphicus or hollandicus or skylark or alauda or tern or sterna or teal or crecca or oystercatcher or haematopus or ostralegus or shrew or shrews or sorex or araneus or

crocidura or russula or european mole or talpa or chiroptera or bat or bats or eptesicus or serotinus or myotis or dasycneme or daubentonii or pipistrelle or pipistrellus or cat or cats or felis or catus or feline or dog or dogs or canis or canine or canines or otter or otters or lutra or badger or badgers or meles or fitchew or fitch or foumart or foulmart or ferrets or ferret or polecat or polecats or mustela or putorius or weasel or weasels or fox or foxes or vulpes or common seal or phoca or vitulina or grey seal or halichoerus or horse or horses or equus or equine or equidae or donkey or donkeys or mule or mules or pig or pigs or swine or swines or hog or hogs or boar or boars or porcine or piglet or piglets or sus or scrofa or llama or llamas or lama or glama or deer or deers or cervus or elaphus or cow or cows or bos taurus or bos indicus or bovine or bull or bulls or cattle or bison or bisons or sheep or sheeps or ovis aries or ovine or lamb or lambs or mouflon or mouflons or goat or goats or capra or caprine or chamois or rupicapra or leporidae or lagomorpha or lagomorph or rabbit or rabbits or oryctolagus or cuniculus or laprine or hares or lepus or rodentia or rodent or rodents or murinae or mouse or mice or mus or musculus or murine or woodmouse or apodemus or rat or rats or rattus or norvegicus or guinea pig or guinea pigs or cavia or porcellus or hamster or hamsters or mesocricetus or cricetulus or cricetus or gerbil or gerbils or jird or jirds or meriones or unguiculatus or jerboa or jerboas or jaculus or chinchilla or chinchillas or beaver or beavers or castor fiber or castor canadensis or sciuridae or squirrel or squirrels or sciurus or chipmunk or chipmunks or marmot or marmots or marmota or suslik or susliks or spermophilus or cynomys or cottonrat or cottonrats or sigmodon or vole or voles or microtus or myodes or glareolus or primate or primates or prosimian or prosimians or lemur or lemurs or lemuridae or loris or bush baby or bush babies or bushbaby or bushbabies or galago or galagos or anthropoidea or anthropoids or simian or simians or monkey or monkeys or marmoset or marmosets or callithrix or cebuella or tamarin or tamarins or saguinus or leontopithecus or squirrel monkey or squirrel monkeys or saimiri or night monkey or night monkeys or owl monkey or owl monkeys or douroucoulis or aotus or spider monkey or spider monkeys or ateles or baboon or baboons or papio or rhesus monkey or macaque or macaca or mulatta or cynomolgus or fascicularis or green monkey or green monkeys or chlorocebus or vervet or vervets or pygerythrus or hominoidea or ape or apes or hylobatidae or gibbon or gibbons or siamang or siamangs or nomascus or symphalangus or hominidae or orangutan or orangutans or pongo or chimpanzee or chimpanzees or pan troglodytes or bonobo or bonobos or pan paniscus or gorilla or gorillas or troglodytes)

25 #18 or #19 or #20 or #21 or #22 or #23 or #24

24 TS=(Baduanjin)

23 TS=(Yijinjing)

22 TS=(Five-animal exercises or Wuqinxi)

21 TS=(Qigong)

20 TS=(Tai Chi or tai ji or taijiquan or taichi)

19 TS=(mind-body exercise)

18 TS=(traditional Chinese exercise)

17 #1 or #2 or #3 or #4 or #5 or #6 or #7 or #8 or #9 or #10 or #11 or #12 or #13 or #14 or #15 or #16
16 TS=(akureyri disease)
15 TS=(iceland disease)
14 TS=(Yuppie)
13 TS=(PVFS)
12 TS=(CFIDS)
11 TS=(royal free disease)
10 TS=(epidemic neuromyasthenia)
9 TS=(systemic exertion intolerance)
8 TS=(chronic Near/3 Epstein Barr)
7 TS=(chronic NEAR/3 mononucleosis)
6 TS=(fatigue-fibromyalgia)
5 TS=(CFS)
4 TS=(myalgic near/3 encephalomyelitis)
3 TS=(fatigue near/3 syndrome)
2 TS=(chronic Near/3 fatigue)
1 TS=(chronic fatigue syndrome)

China Knowledge Resource Integrated Database (CNKI，资源范围:总库；同义词扩展；更新时间:不限)

#1 (主题=慢性疲劳综合征) OR (主题=肌痛性脑脊髓炎) OR (主题=疲劳) OR (主题=乏力) OR (主题=肌痛) OR (主题=肌无力)

#2 (主题=功法) OR (主题=太极) OR (主题=气功) OR (主题=五禽戏) OR (主题=八段锦) OR (主题=易筋经)

#2 (主题=随机) OR (主题=对照) OR (主题=临床)

#3 1 AND 2 AND 3

Wanfang Data Information

主题:(“慢性疲劳综合征”or “肌痛性脑脊髓炎” or “疲劳”or “乏力” or “肌痛” or “肌无力”) and 主题:(“随机”or“对照”or “临床”) and 主题:(“功法”or “太极” or “气功”or “八段锦” or “易筋经” or “五禽戏”)

Weipu Database for Chinese Technical Periodicals

(((((((题名或关键词=慢性疲劳综合征 OR 题名或关键词=肌痛性脑脊髓炎) OR 题名或关键词=疲劳) OR 题名或关键词=乏力) OR 题名或关键词=肌痛) OR 题名或关键词=肌无力) AND (((((题名或关键词=功法 OR 题名或关键词=太极) OR 题名或关键词=气功) OR 题名或关键词=八段锦) OR 题名或关键词=易筋经) OR 题名或关键词=五禽戏)) AND ((题名或关键词=随机 OR 题名或关键词=对照) OR 题名或关键词=临床))

Risk of bias assessment (Figure S1)

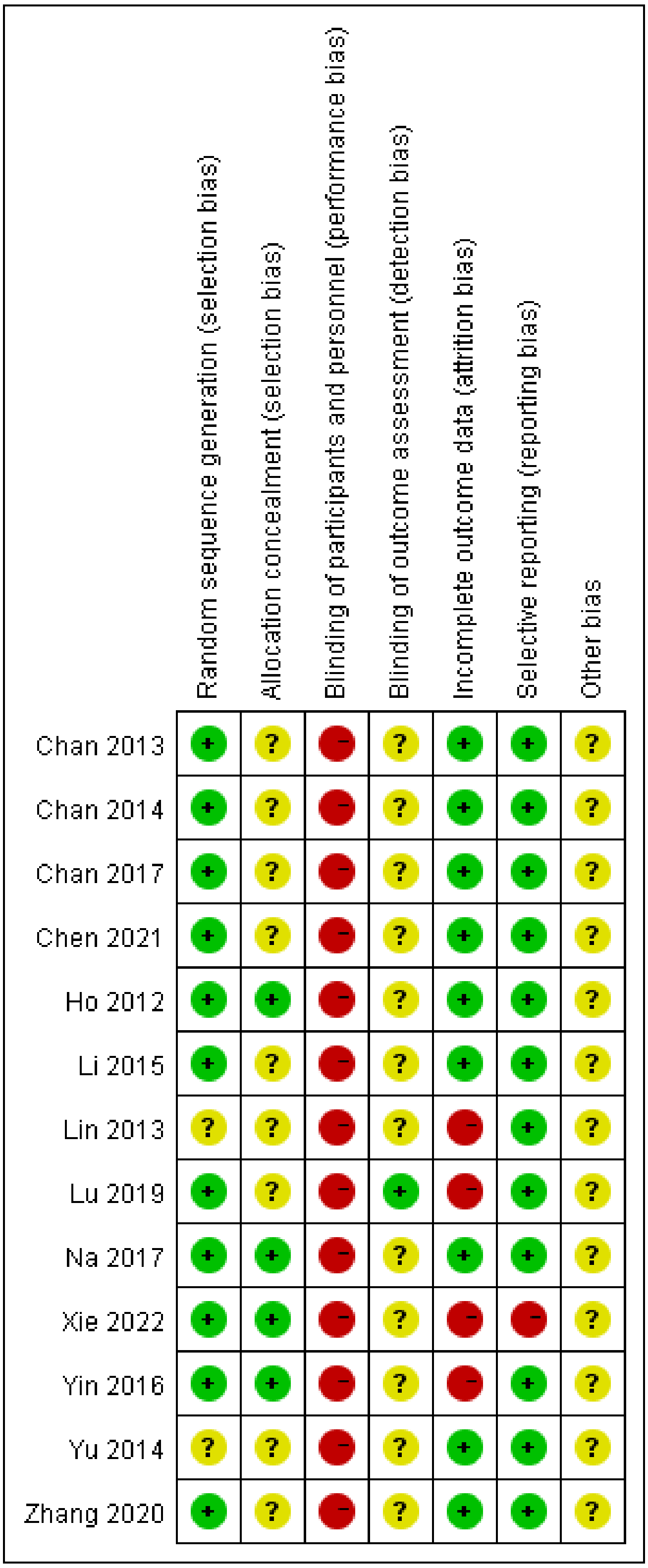

The results of subgroup analyses (Figure S2-S5)

Figure S2 TCME with background therapy versus TCME without background therapy.

Study or Subgroup	TCME Mean	TCME SD	TCME Total	Passive control Mean	Passive control SD	Passive control Total	Weight	Std. Mean Difference IV, Random, 95% CI
**1 TCME with background therapy**								
Lin 2013	62.6	28.87	27	49.1	28.41	26	8.2%	0.46 [-0.08, 1.01]
Yu 2014	1.14	1.87	42	0.22	1.84	42	10.2%	0.49 [0.06, 0.93]
Na 2017	4.15	1.22	30	2.45	1.2	30	7.9%	1.39 [0.82, 1.95]
Lu 2019	6.5	2.63	24	5.18	2.56	23	7.7%	0.50 [-0.08, 1.08]
Zhang 2020	3.57	1.97	39	1.9	1.82	39	9.6%	0.87 [0.41, 1.34]
Chen 2021	20.88	7.86	34	10.61	8.98	36	8.8%	1.20 [0.69, 1.71]
**Subtotal (95% CI)**			**196**			**196**	**52.4%**	**0.81 [0.50, 1.12]**
Heterogeneity: Tau² = 0.08; Chi² = 10.97, df = 5 (P = 0.05); I² = 54%								
Test for overall effect: Z = 5.13 (P < 0.00001)								
**2 TCME without background therapy**								
Ho 2012	18.3	9.77	33	7.6	8.47	31	8.5%	1.15 [0.62, 1.69]
Chan 2013	13.1	12.52	72	6.6	9.14	65	12.1%	0.59 [0.24, 0.93]
Chan 2014	11.8	11.61	75	4.1	9.93	75	12.4%	0.71 [0.38, 1.04]
Li 2015	20.22	7.32	22	5.29	9.33	24	6.3%	1.74 [1.05, 2.43]
Yin 2016	4.19	3.33	27	1.62	3.68	30	8.4%	0.72 [0.18, 1.26]
**Subtotal (95% CI)**			**229**			**225**	**47.6%**	**0.90 [0.57, 1.24]**
Heterogeneity: Tau² = 0.09; Chi² = 10.75, df = 4 (P = 0.03); I² = 63%								
Test for overall effect: Z = 5.27 (P < 0.00001)								
**Total (95% CI)**			**425**			**421**	**100.0%**	**0.85 [0.64, 1.07]**
Heterogeneity: Tau² = 0.07; Chi² = 21.73, df = 10 (P = 0.02); I² = 54%								
Test for overall effect: Z = 7.76 (P < 0.00001)								
Test for subgroup differences: Chi² = 0.15, df = 1 (P = 0.69), I² = 0%								

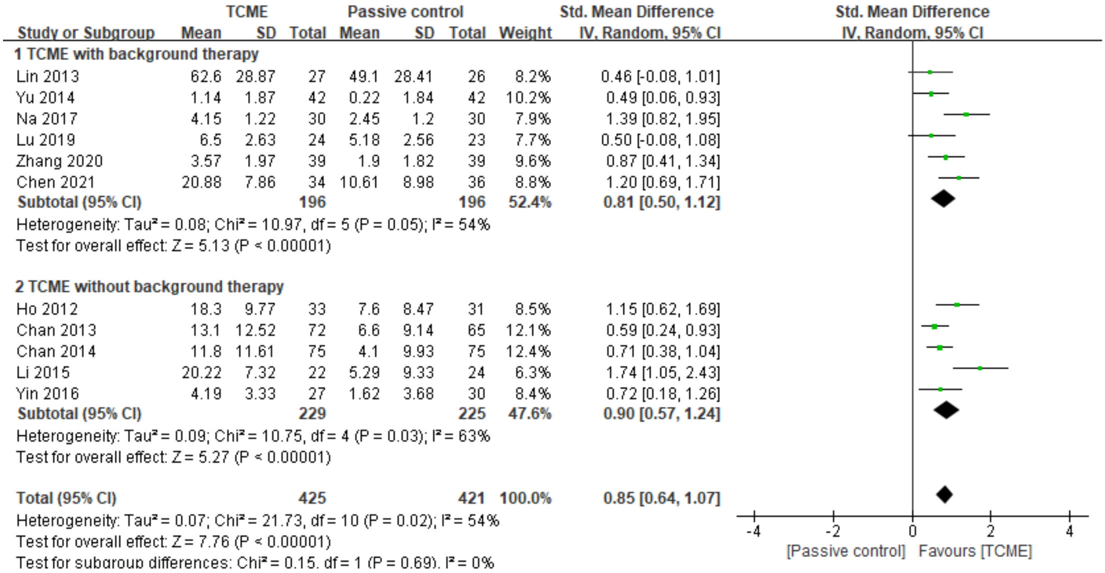

Figure S3 Pharmacological intervention versus non-pharmacological intervention.

Study or Subgroup	TCME Mean	TCME SD	TCME Total	Passive control Mean	Passive control SD	Passive control Total	Weight	Std. Mean Difference IV, Random, 95% CI
**1 Pharmacological interventions**								
Yu 2014	1.14	1.87	42	0.22	1.84	42	19.2%	0.49 [0.06, 0.93]
Zhang 2020	3.57	1.97	39	1.9	1.82	39	18.2%	0.87 [0.41, 1.34]
Chen 2021	20.88	7.86	34	10.61	8.98	36	16.8%	1.20 [0.69, 1.71]
**Subtotal (95% CI)**			**115**			**117**	**54.2%**	**0.84 [0.44, 1.24]**
Heterogeneity: Tau² = 0.07; Chi² = 4.38, df = 2 (P = 0.11); I² = 54%								
Test for overall effect: Z = 4.09 (P < 0.0001)								
**2 Non-pharmacological interventions**								
Lin 2013	62.6	28.87	27	49.1	28.41	26	15.8%	0.46 [-0.08, 1.01]
Na 2017	4.15	1.22	30	2.45	1.2	30	15.2%	1.39 [0.82, 1.95]
Lu 2019	6.5	2.63	24	5.18	2.56	23	14.8%	0.50 [-0.08, 1.08]
**Subtotal (95% CI)**			**81**			**79**	**45.8%**	**0.78 [0.19, 1.37]**
Heterogeneity: Tau² = 0.19; Chi² = 6.56, df = 2 (P = 0.04); I² = 70%								
Test for overall effect: Z = 2.60 (P = 0.009)								
**Total (95% CI)**			**196**			**196**	**100.0%**	**0.81 [0.50, 1.12]**
Heterogeneity: Tau² = 0.08; Chi² = 10.97, df = 5 (P = 0.05); I² = 54%								
Test for overall effect: Z = 5.13 (P < 0.00001)								
Test for subgroup differences: Chi² = 0.02, df = 1 (P = 0.88), I² = 0%								

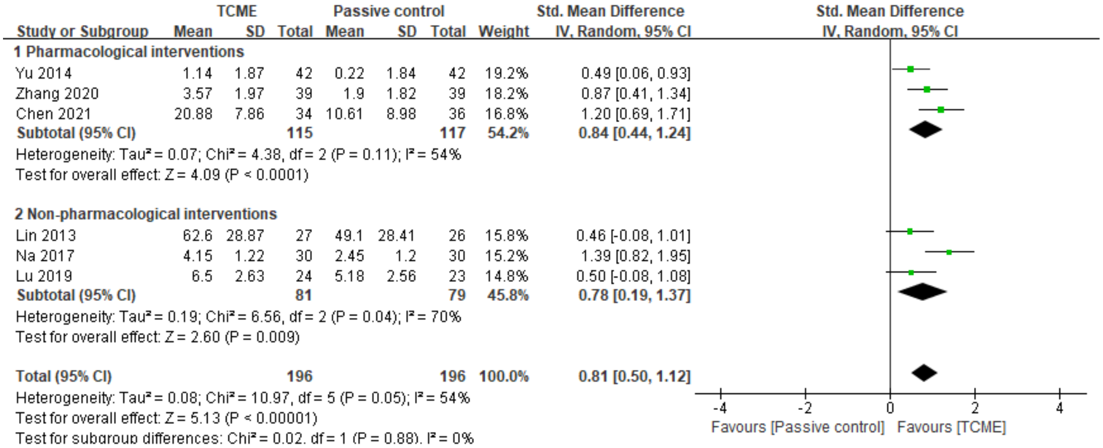

Figure S4 Qigong versus Tai Chi.

Study or Subgroup	TCME Mean	TCME SD	TCME Total	Passive control Mean	Passive control SD	Passive control Total	Weight	Std. Mean Difference IV, Random, 95% CI
**1 Qigong**								
Yu 2014	1.14	1.87	42	0.22	1.84	42	19.2%	0.49 [0.06, 0.93]
Na 2017	4.15	1.22	30	2.45	1.2	30	15.2%	1.39 [0.82, 1.95]
Lu 2019	6.5	2.63	24	5.18	2.56	23	14.8%	0.50 [-0.08, 1.08]
**Subtotal (95% CI)**			**96**			**95**	**49.2%**	**0.78 [0.22, 1.34]**
Heterogeneity: Tau² = 0.18; Chi² = 6.90, df = 2 (P = 0.03); I² = 71%								
Test for overall effect: Z = 2.71 (P = 0.007)								
**2 Tai Chi**								
Lin 2013	62.6	28.87	27	49.1	28.41	26	15.8%	0.46 [-0.08, 1.01]
Zhang 2020	3.57	1.97	39	1.9	1.82	39	18.2%	0.87 [0.41, 1.34]
Chen 2021	20.88	7.86	34	10.61	8.98	36	16.8%	1.20 [0.69, 1.71]
**Subtotal (95% CI)**			**100**			**101**	**50.8%**	**0.86 [0.46, 1.25]**
Heterogeneity: Tau² = 0.06; Chi² = 3.73, df = 2 (P = 0.16); I² = 46%								
Test for overall effect: Z = 4.20 (P < 0.0001)								
**Total (95% CI)**			**196**			**196**	**100.0%**	**0.81 [0.50, 1.12]**
Heterogeneity: Tau² = 0.08; Chi² = 10.97, df = 5 (P = 0.05); I² = 54%								
Test for overall effect: Z = 5.13 (P < 0.00001)								
Test for subgroup differences: Chi² = 0.05, df = 1 (P = 0.83), I² = 0%								

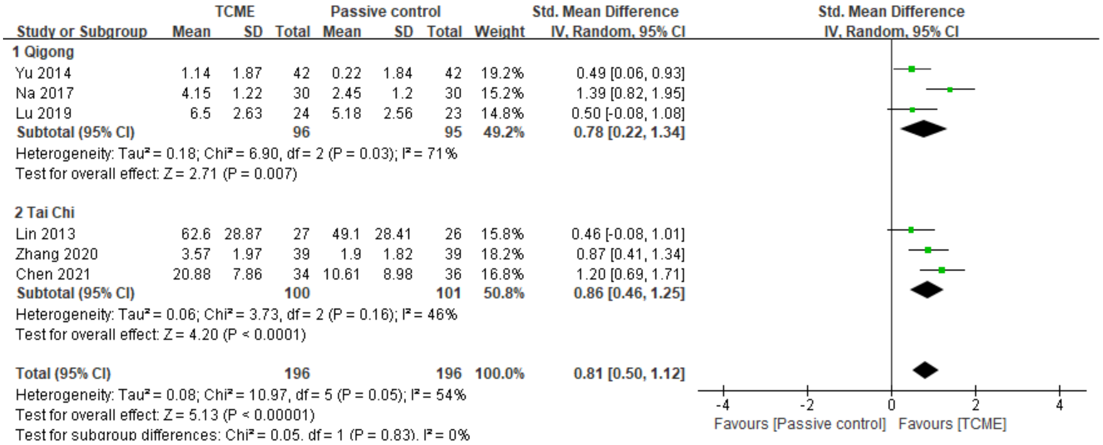

Figure S5 Physical fatigue versus mental fatigue.

Study or Subgroup	TCME Mean	TCME SD	TCME Total	Passive control Mean	Passive control SD	Passive control Total	Weight	Std. Mean Difference IV, Random, 95% CI
**1 Physical fatigue**								
Ho 2012	12.1	5.73	33	4.4	5.53	31	11.4%	1.35 [0.80, 1.90]
Chan 2013	8.8	7.38	72	3.8	5.41	65	14.2%	0.76 [0.41, 1.11]
Chan 2014	7.5	7.38	75	2.4	6.02	75	14.4%	0.75 [0.42, 1.08]
Li 2015	14.45	5.02	22	3.53	5.5	24	9.2%	2.03 [1.31, 2.76]
**Subtotal (95% CI)**			**202**			**195**	**49.3%**	**1.14 [0.66, 1.61]**
Heterogeneity: Tau² = 0.17; Chi² = 13.15, df = 3 (P = 0.004); I² = 77%								
Test for overall effect: Z = 4.70 (P < 0.00001)								
**2 Mental fatigue**								
Ho 2012	6.1	4.57	33	3.1	3.87	31	12.0%	0.70 [0.19, 1.20]
Chan 2013	4.4	5.75	72	2.8	4.89	65	14.3%	0.30 [-0.04, 0.63]
Chan 2014	4.2	5.15	75	1.6	4.98	75	14.5%	0.51 [0.19, 0.84]
Li 2015	9.87	3.69	22	3	4.86	24	9.9%	1.56 [0.89, 2.22]
**Subtotal (95% CI)**			**202**			**195**	**50.7%**	**0.69 [0.27, 1.11]**
Heterogeneity: Tau² = 0.13; Chi² = 11.26, df = 3 (P = 0.01); I² = 73%								
Test for overall effect: Z = 3.22 (P = 0.001)								
**Total (95% CI)**			**404**			**390**	**100.0%**	**0.91 [0.59, 1.24]**
Heterogeneity: Tau² = 0.16; Chi² = 31.39, df = 7 (P < 0.0001); I² = 78%								
Test for overall effect: Z = 5.52 (P < 0.00001)								
Test for subgroup differences: Chi² = 1.95, df = 1 (P = 0.16), I² = 48.7%								

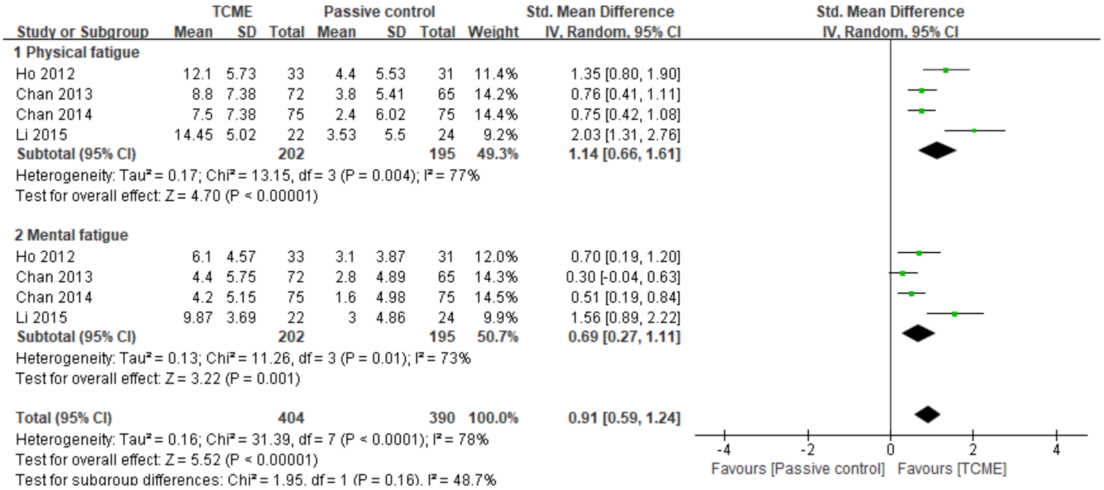

Excluded studies with reasons for exclusion at full-text screening stage (Table S1)

Reference	Reason for exclusion
Liu 2010 a[1]	No relevant outcomes reported
Liu 2010 b[2]	No relevant outcomes reported
Jing 2012[3]	Not chronic fatigue syndrome
Saganha 2012[4]	Not chronic fatigue syndrome
Liao 2013[5]	Not randomized controlled trial
Geng 2013[6]	Not chronic fatigue syndrome
Maddali 2016[7]	Not chronic fatigue syndrome
Qian 2016[8]	Ineligible comparison
Zhu 2016[9]	Not chronic fatigue syndrome
Yu 2018[10]	Not chronic fatigue syndrome
Chan 2019[11]	Not randomized controlled trial
Li 2020[12]	Not chronic fatigue syndrome
Xie 2020[13]	Duplicate publication
Xu 2020[14]	Not chronic fatigue syndrome
Li 2022[15]	Not randomized controlled trial
Xie 2022 a[16]	Duplicate publication
Xie 2022 b[17]	Duplicate publication

[1] Liu CZ, Lei B. A Randomized Controlled Trial of Manipulation for Chronic Fatigue Syndrome. Liaoning Journal of Traditional Chinese Medicine. 2010,37(02): 272-273.

[2] Liu CZ, Lei B. Effect of Tuina on oxygen free radicals metabolism in patients with chronic fatigue syndrome. Chinese Acupuncture & Moxibustion. 2010,30(11): 946-948.

[3] Jing JH, Liu ZK. A study on the effect of different physical exercises on the basic physical Fitness of fatigue Sub-healthy College students. Contemporary Sports Technology. 2012,2(22): 13-14.

[4] Saganha JP, Doenitz C, Greten T, Efferth T, Greten HJ. Qigong therapy for physiotherapists suffering from burnout: a preliminary study. Zhong Xi Yi Jie He Xue Bao. 2012 Nov;10(11):1233-9.

[5] Liao Y, Lin Y, Zhang C, et al. The Effects of Baduanjin on Life Quality in People of Fatigue-predominant Sub health. Proceedings of the Symposium on the Research and Development of Traditional Chinese Medicine Health Food and the Method of Health Preservation in Traditional Chinese Medicine. 2013:73-78.

[6] Geng YQ. Effects Research of Baduanjin & Five-Notes Music on the Mental Sub-Health State. Doctorate's thesis, Nanjing University of Chinese Medicine, Nanjing, China, 2013.

[7] Maddali BS, Paoletti G, Calà M, Del RA, EL Aoufy K, Mikhaylova S. Efficacy of rehabilitation with Tai Ji Quan in an Italian cohort of patients with Fibromyalgia Syndrome. Complementary Therapies in Clinical Practice. 2016,24: 109-115.

[8] Qian LF, Yao Q, Zhu GF. Effect of channel points massage combined with Yijinjing exercising on chronic fatigue syndrome with sleep disorder. Chinese Journal of General Practice. 2016,14(11): 1886-1888.
[9] Zhu GF. Experimental Study of Traditional College Students Sub-health Care Intervention. Contemporary Sports Technology. 2016,6(31): 85-87.
[10] Yu JL. Eight sections of brocade to college students cervical vertebra study of health interventions. Master's thesis, Hunan Normal University, Changsha, China, 2018.
[11] Chan JSM, Ng SM, Yuen LP, Chan CLW. Qigong exercise for chronic fatigue syndrome. Int Rev Neurobiol. 2019;147:121-153.
[12] Li J, Yan XE, Zhang Y, et al. Intervention research of Baduanjin exercise in relieving college students with mental fatigue. China Medical Herald. 2020,17(16): 112-115.
[13] Xie FF, Wang WJ, Guan C, Cheng ZJ, Zhou WQ, Yao F. Clinical study of the Prolong Life with Nine Turn Method on CFS fatigue and quality of life. Lishizhen Medicine And Materia Medica Research. 2020,31(12): 2951-2955.
[14] Xu SD, Kong LJ, Zhu QG, et al. Clinical research of Daoyin Gongfa in preventing the transformation of qi deficiency into chronic fatigue syndrome. China Journal of Traditional Chinese Medicine and Pharmacy. 2020,35(12): 6042-6045.
[15] Li YY, Wu K, HU XJ, et al. Altered Effective Connectivity of Resting-State Networks by Tai Chi Chuan in Chronic Fatigue Syndrome Patients: A Multivariate Granger Causality Study. Front Neurol. 2022,13:858833.
[16] Xie FF, Guan C, Gu YJ, You YL, Yao F. Effects of the Prolong Life With Nine Turn Method (Yan Nian Jiu Zhuan) Qigong on Brain Functional Changes in Patients With Chronic Fatigue Syndrome in Terms of Fatigue and Quality of Life. Front Neurol. 2022,13:866424.
[17] Xie FF, Guan C, Cheng ZJ, Gu YJ, Yao F. Amplitude of Low-Frequency Fluctuations Study on the Effect of YannianJiuzhuan Method on the Quality of Life of Patients with Chronic Fatigue Syndrome. Journal of Clinical Radiology. 2022,41(08):1553-1558.
